# Mesenchymal Stem Cells Carrying Viral Fusogenic Protein p14 to Treat Solid Tumors by Inducing Cell–Cell Fusion and Immune Activation

**DOI:** 10.34133/research.0594

**Published:** 2025-01-27

**Authors:** Yao Wang, Xunlei Pang, Ruirui Li, Jiuzhou Chen, Chen Wen, Huihuang Zhu, Tingyu Long, Jianjie Li, Lijun Zheng, Youcai Deng, Junnian Zheng, Bo Xu

**Affiliations:** ^1^ Cancer Institute, Xuzhou Medical University, Xuzhou, Jiangsu 221004, China.; ^2^ Center of Clinical Oncology, The Affiliated Hospital of Xuzhou Medical University, Xuzhou, Jiangsu 221002, China.; ^3^ Jiangsu Center for the Collaboration and Innovation of Cancer Biotherapy, Xuzhou Medical University, Xuzhou, Jiangsu 221004, China.; ^4^ Department of Gastroenterology, The Affiliated Hospital of Xuzhou Medical University, Xuzhou, Jiangsu 221004, China.; ^5^ Department of Radiation Oncology, the Affiliated Huaian No.1 People’s Hospital of Nanjing Medical University, Huaian, Jiangsu, China.; ^6^ Department of Clinical Hematology, College of Pharmacy and Laboratory Medicine Science, Army Medical University, Chongqing 400038, China.

## Abstract

**Background:** Chimeric antigen receptor (CAR)-based immune cell therapies attack neighboring cancer cells after receptor recognition but are unable to directly affect distant tumor cells. This limitation may contribute to their inefficiency in treating solid tumors, given the restricted intratumoral infiltration and immunosuppressive tumor microenvironment. Therefore, cell–cell fusion as a cell-killing mechanism might develop a novel cytotherapy aimed at improving the efficacy against solid tumors. **Methods:** We constructed a fusogenic protein, fusion-associated small transmembrane (FAST) p14 of reptilian reovirus, into cancer cells and mesenchymal stem cells (MSCs), which cocultured with various colon cancer cells and melenoma cells to validate its ability to induce cell fusion and syncytia formation. RNA sequencing, quantitative reverse transcription polymerase chain reaction, and Western blot were performed to elucidate the mechanism of syncytia death. Cell viability assay was employed to assess the killing effects of MSCs carrying the p14 protein (MSCs-p14), which was also identified in the subcutaneous tumor models. Subsequently, the Tet-On system was introduced to enhance the controllability and safety of therapy. **Results:** Cancer cells incorporated with fusogenic protein p14 FAST from reovirus fused together to form syncytia and subsequently died through apoptosis and pyroptosis. MSCs-p14 cocultured with different cancer cells and effienctly induced cancer cell fusion and caused widespread cancer cell death in vitro. In mouse tumor models, mMSCs-p14 treatment markedly suppressed tumor growth and also enhanced the activity of natural killer cells and macrophages. Controllability and safety of MSCs-p14 therapy were further improved by introducing the tetracycline-controlled transcriptional system. **Conclusion:** MSC-based cytotherapy carrying viral fusogenic protein in this study kills cancer cells by inducing cell–cell fusion. It has demonstrated definite efficacy in treating solid tumors and is worth considering for clinical development.

## Introduction

Genetically engineered cells are widely used in antitumor biotherapies [1]. Chimeric antigen receptor (CAR) T cell therapy, one of the most successful cytotherapies, has achieved great success in the treatment of hematologic malignancies [2,3]. However, its efficacy against solid tumors remains unsatisfactory. CAR T cells, developed based on the CARs and their natural cytotoxicity, are limited to kill only adjacent tumor cells after recognition, without directly affecting distant tumor cells. This limitation may be a fundamental reason for the suboptimal efficacy of CAR T cell therapy against solid tumors, especially in the context of an immunosuppressive tumor microenvironment and limited tumor infiltration [4]. Therefore, we propose introducing a novel cell-killing mechanism, cell–cell fusion, to develop an antitumor cell therapy aimed at broadly and distantly killing tumor cells, thereby improving therapeutic efficacy against solid tumors.

Cell fusion is an important cellular process in which multiple uninucleate cells merge their plasma membranes to form a single multinucleate cell, known as a syncytium [5]. Cell fusion plays a critical role in many normal physiological processes, including fertilization, placenta formation, and muscle development [6–8]. It is also involved in various pathological conditions, including infections by bacteria [9,10], parasites [11,12], and viruses [13,14]. The cellular structure of syncytia becomes unstable. Its formation leads to a cytopathic effect, finally resulting in cell death [15,16]. This syncytial cytopathic effect caused by cell fusion presents an ideal cell-killing mechanism for developing biotherapies against solid tumor, because the fused cells will form a single entity that continuously affects surrounding cells, achieving a broad and long-range antitumor cell-killing effect. Additionally, the immunogenic cell death induced by cell fusion can further mobilize the body’s immune response to participate in antitumor activity.

As we previously reported, enhancing the syncytial characteristic of oncolytic herpes viruses by inserting an E-cadherin coding gene improved their therapeutic efficacy against glioblastoma to a certain extent by inducing cell–cell fusion [17]. Wong et al. [18] incorporated the p14 fusion-associated small transmembrane (FAST) protein from reptilian reovirus into oncolytic adenoviruses, promoting virus replication and spreading. However, in the immunocompetent mouse models of breast cancer and lung cancer, the therapitic benefit from this modification was not as significant as it was in vitro. The rapid immune clearance of the oncolytic virus after administration may reasonably explain why the effect improvement was significant in vitro but slight in vivo. Compared to oncolytic viruses, engineered mesenchymal stem cells (MSCs) appear to be a better vehicle for delivering fusogenic proteins to treat cancers because of their lower immunogenicity, which allows for longer persistence in action [19]. Their lower toxicity and tumor-homing characteristics make them more favorable for achieving intravenous administration [20]. Additionally, numerous clinical trials have thoroughly validated the safety and reliability of MSCs as gene therapy vectors [21–23].

In this study, we integrated a fusogenic protein, FAST p14 of reptilian reovirus, into colon cancer and melanoma cells, demonstrating its ability to induce cell fusion and syncytia formation with both itself and adjacent p14-negative cancer cells. This process led to cell death by activating apoptosis and pyroptosis. Subsequently, MSCs were employed as effector cells to deliver the p14 protein (MSCs-p14). MSCs-p14 initiated cell fusion and effectively induced widespread cancer cell death in vitro. In the subcutaneous tumor models of colon cancer and melanoma, MSCs-p14 treatment significantly reduced tumor growth, highlighting the therapeutic potential of MSCs-p14 in vivo.

## Results

### p14 expression induced extensive cell–cell fusion of cancer cells

**Fig. 1.**
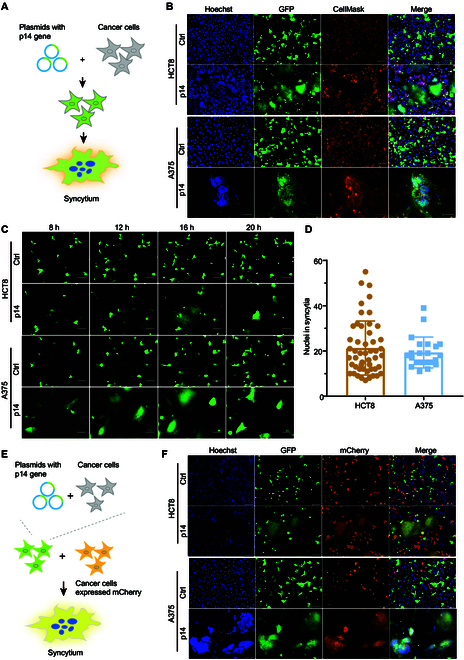
Cancer cell fusion induced by p14 protein expression. Plasmids of PCDH-GFP or PCDH-GFP-p14 were transfected into HCT8 and A375 cells separately and expressed for 16 h. Then, cells were stained with CellMask (far red) for the cell membrane and Hoechst 33342 (blue) for the nuclei, and imaged with a fluorescence microscope. (A) Diagram illustrating p14 protein expression inducing cell–cell fusion. (B) The protein expression of p14 induced the fusion of cancer cells of HCT8 and A375. Compared to blank cells, multinucleated syncytia were formed within the p14-expressing cells. (C) Time-lapse images of syncytia formation and enlarging process. Images were taken at 8, 12, 16, and 20 h after transfection. Scale bar, 100 μm. (D) Number of nuclei in syncytia of HCT8 and A375 cells. Sixteen hours after transfection, 20 fields were randomly selected for imaging with fluorescence microscopy after nuclei staining. Nuclei in syncytia were counted manually. (E) Diagram of p14-positive cells fused with p14-negative cells. (F) Plasmids of PCDH-GFP or PCDH-GFP-p14 were transfected into cancer cells and subsequently cocultured with cancer cells expressing mCherry protein for further 20 h. The images of syncytia that were GFP and mCherry double positive were collected.

For developing a novel antitumor cytotherapy that kills cancer cells by inducing cell–cell fusion, we proposed using the FAST protein p14 from reptilian reovirus, one of the most efficient viral fusogenic proteins, to induce cancer cell fusion. To study whether the expression of p14 was sufficient to induce cancer cell fusion, we transfected various cancer cell lines, including 4 colon cancer cell lines (HCT8, LOVO, SW480, and MC38) and 4 melanoma cell lines (A375, MV3, M14, and B16F10) with the p14-expressing plasmid (PCDH-GFP-p14) or the empty vector (PCDH-GFP) as control. Both plasmids carried a green fluorescent protein (GFP) reporter gene (Fig. 1A). The results showed that 16 h after transfection, multinucleated syncytia were formed in all the cell lines transfected with p14-expressing plasmid but not in the control groups. Staining of cell membrane and nuclei revealed that the p14-induced syncytia were characterized as areas with multiple nuclei clustered but lacking obvious cell membrane structure separating them (Fig. 1B and Fig. S1A and B). Furthermore, time-lapse images and videos were taken to study the process of p14-induced cell–cell fusion. HCT8 and A375 cancer cells were transfected with plasmids of either PCDH-GFP-p14 plasmid or PCDH-GFP as control. The results clearly showed the initiation of syncytia formation, their enlargement, and subsequent shrinking over time. With the aid of nuclear staining, we observed that the p14-induced cell fusion initiated at around 8 h after transfection, when cells with 2 nuclei first appeared. The syncytia then continued to grow as more cell nuclei gathered into the central region of the syncytia, reaching the maximum size at around 16 h after transfection. The syncytia were shrunken 20 h after transfection and lysed successively (Fig. 1C, Fig. S1C and D, and Movies S1 and S2). At 16 h after transfection, aggregated nuclei in syncytia from 20 fields were counted. The results showed that each syncytium contained an average of 21 and 19 nuclei within HCT8 and A375 cells, respectively (Fig. 1D). We next investigated whether p14-expressing cells could fuse with p14-negative cells. For this purpose, HCT8 and A375 cells transfected with PCDH-GFP-p14 plasmid were used as effector cells and cocultured with the corresponding target cells transfected with empty vector (PCDH-mCherry). At 16 h after coculture, GFP and mCherry double-positive syncytia were observed, indicating that p14-expressing cells had fused with p14-negative cells (Fig. 1E and F and Fig. S1E and F). Taken together, these data suggest that the effector cells expressing p14 can efficiently fuse with different types of cancer cells, indicating that p14 is suitable for developing a broad-spectrum antitumor therapy that kills tumor cells by inducing cell–cell fusion.

### Apoptosis and pyroptosis occurred within the syncytia formed by p14 induction

**Fig. 2.**
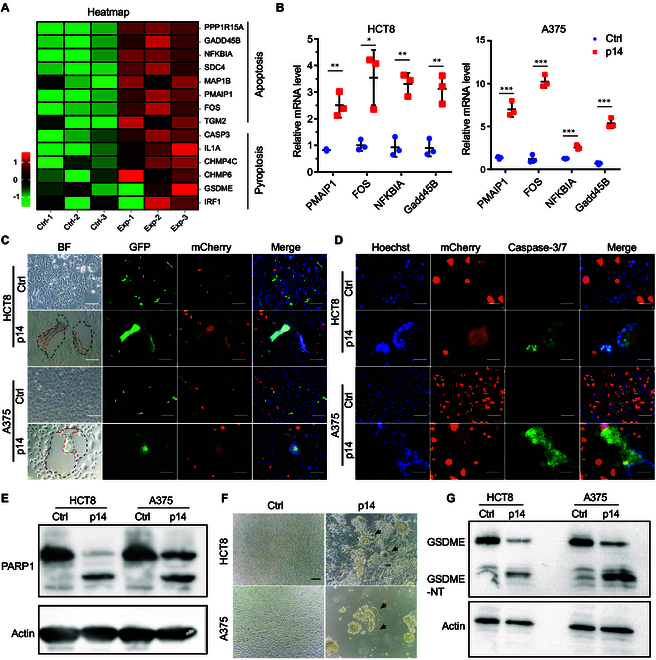
p14 protein induced cell death through apoptosis and pyroptosis. (A) Apoptosis and pyroptosis were activated at the late stage of cell fusion. In RNA-seq assay, HCT8 cells were transfected with PCDH-GFP or PCDH-GFP-p14 plasmids. Ten million cells were harvested with TRIzol reagent 20 h after transfection. Heatmap showed up-regulated apoptosis and pyroptosis-related genes. (B) The mRNA level of apoptosis-related genes in HCT8 and A375 was analyzed by qPCR. Data were analyzed and presented as mean ± SD (*n* = 3 technical replicates). Sample comparisons were performed using multiple *t* test corrected by FDR. For HCT8 cells, Ctrl versus p14 in PMAIP1, **P = 0.003; in FOS, *P = 0.014; in NFKBIA, **P = 0.002; in Gadd45B, **P = 0.003. For A375 cells, Ctrl versus p14 in 4 genes, ***P < 0.001. (C) Syncytia were shrunk and died at the late stage of cell fusion. Plasmids of PCDH-GFP or PCDH-GFP-p14 were transfected into cancer cells of HCT8 and A375 and cocultured with corresponding cells expressing mCherry protein. Twenty hours after transfection, shrinking was observed. The area outlined by the black dashed line indicated the unfolded syncytia, and those outlined by the red dashed line indicated the shape of shrunk syncytia. (D) Plasmids of PCDH-mCherry and PCDH-mCherry-p14 were transfected into cells. Twenty hours after transfection, cells were labeled with probe of caspase-3/7 and nuclei were stained with Hoechst 33342 and subsequently imaged with a fluorescence microscope. (E) Poly(adenosine diphosphate-ribose) polymerase 1 (PARP1) was cleaved in the cell–cell fusion. HCT8 and A375 were transfected with PCDH-GFP or PCDH-GFP-p14 plasmids, and the cells were harvested 20 h after transfection. Fragments of PARP1 were measured by immunoblotting. (F) Syncytia shrinkage and extracellular pyroptosis vesicles were imaged in p14-expressing cells. Vesicles were indicated by black arrows. (G) GSDME cleavage was analyzed by immunoblotting 20 h after transfection.

**Fig. 3.**
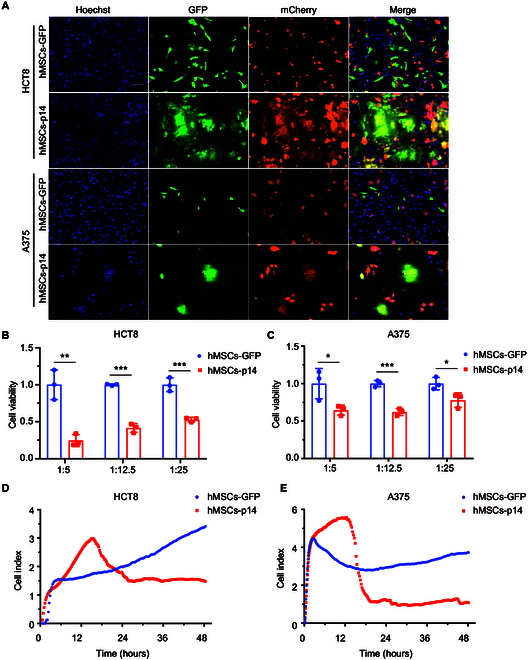
MSCs carrying p14 protein induced cell–cell fusion with human cancer cells. Plasmids of PCDH-GFP or PCDH-GFP-p14 were transfected into hMSCs separately. Cells were harvested with trypsin 8 h after transfection, then mixed with HCT8 and A375 expressing mCherry, and cocultured for further 18 h. (A) Images of hMSCs-p14 induced cell–cell fusion and syncytium formation. Nuclei were stained with Hoechst 33342. Scale bar, 100 μm. (B and C) CCK8 analysis of killing effects of hMSCs-p14 on HCT8 and A375. Human MSCs were transfected with plasmids of PCDH-GFP and PCDH-GFP-p14 and mixed with cancer cells at 8 h after transfection at the ratios of 1:5, 1:12.5, and 1:25. Forty-eight hours after coculture, the cells were added with CCK8 and the absorbance at 450 nm was measured. The data were analyzed and presented as mean ± SD (*n* = 3 well replicates). Sample comparisons were performed using multiple *t* test corrected by FDR. For HCT8 cells, hMSCs-14 versus hMSCs-GFP at a ratio of 1:5, **P = 0.004; at ratio of 1:12.5, ***P < 0.001; at ratio of 1:25, **P = 0.001. For A375 cells, hMSCs-14 versus hMSCs-GFP at a ratio of 1:5, *P = 0.044; at ratio of 1:12.5, ***P < 0.001; at ratio of 1:25, *P = 0.040. (D and E) xCELLigence RTCA technology was used to measure the activity of fusion and killing of hMSCs-p14 on HCT8 and A375 cells. Human MSCs were transfected with plasmids of PCDH-GFP and PCDH-GFP-p14 and mixed with cancer cells 8 h after transfection at a ratio of 1:5. The fusion and killing efficiency was monitored for 48 h.

As mentioned above, p14-induced syncytia shrank and died eventually. To uncover the mechanisms of p14-induced cell–cell fusion leading to cell death, HCT8 cells were transfected with PCDH-GFP-p14 plasmids for 20 h and RNA-sequencing (RNA-seq) analysis was performed to examine the content of differentially expressed genes (DEGs). A total of 126 DEGs [false discovery rate (FDR) < 0.05 in combination with fold change ≥ 2.0] were screened in the p14-transfected group when compared with the control group, including 82 up-regulated genes and 44 down-regulated genes (Fig. S2A and B). Among these genes, several up-regulated genes were related to apoptosis (Fig. 2A). The overrepresentation analysis was used to estimate the functions of the DEGs. We found that apoptosis-related pathway was enriched (Fig. S2C). Likewise, expression datasets were subjected to Gene Set Enrichment Analysis (GSEA). Two gene sets related to apoptosis were significantly enriched: “Apoptosis by Reovirus infection up“ and ”Modulation and signaling of apoptosis”. Both sets showed positive normalized enrichment scores (Fig. S2D). Four apoptosis-related genes (PMAIP1, FOS, NFKBIA, and Gadd45B) were confirmed up-regulated in HCT8, A375, LOVO, and MV3 cells by quantitative polymerase chain reaction (PCR), consistent with RNA-seq data (Figs. 2B and 3 and Fig. S2E). Additionally, when p14-expressing cancer cells were cocultured with p14-negative cells for 20 h, the formed syncytia were shrunk and caspase-3/7 expression was increased in syncytia (Fig. 2C and D and Fig. S4). Subsequently, we checked the expression of apoptosis-related protein PRAP1. Increased cleavage of PRAP1 was observed in HCT8 and A375 cells (Fig. 2E). In addition, we noticed that several pyroptosis-related genes were up-regulated (Fig. 2A). Therefore, we verified whether cell fusion could induce pyroptosis and subsequently led to cancer cell death. Accordingly, we found the numerous vesicles adhered on syncytia at late of cell fusion and increased over time (Fig. 2F). Meanwhile, cleavage of pyroptosis marker protein GSDME was increased (Fig. 2G). Three hours after transfection, Z-DEVD was added to inhibit the cleavage of caspase-3 and pyroptosis. We found that the shrinkage and lysis of p14-induced syncytia were slowed down, indicating the contribution of pyroptosis to the destruction of fused tumor cells (Fig. S5). Thus, syncytia death was related to pyroptosis. GSDME was cleaved by caspase-3, and subsequently, N-GSDME was intercalated into syncytia membrane, disrupting membrane integrity, eventually leading to cell death. Taken together, these data revealed that apoptosis and pyroptosis were activated by cell fusion and led to cancer cell death.

Furthermore, to investigate whether the alterations in the inflammatory response were induced by cell–cell fusion, we performed a detailed analysis of the RNA-sequencing data. This analysis revealed activation of inflammatory pathways and regulation of the transcription levels of caspase family members.(Fig. S6A and B). Quantitative reverse transcription PCR (RT-PCR) was conducted to confirm this finding, showing a significant increase in the transcription of interferon-β (IFN-β), tumor necrosis factor-α (TNF-α), interleukin-1β (IL-1β), IL-12A, and IL-23A after cell–cell fusion (Fig. S6C and D). Additionally, increased secretions of IL-1β and IL-18 were confirmed by enzyme-linked immunosorbent assay (ELISA) assays (Fig. S6E).

### MSCs carrying p14 fused with and killed cancer cells in vitro

MSCs possess unique properties of immune privilege and tumor homing, which make MSCs a promising vehicle for developing cell-based antitumor therapies. To study whether MSCs were feasible for carrying p14 protein to fuse with cancer cells, human umbilical cord blood-derived MSCs (hMSCs) were transfected with p14-expressing plasmid (hMSCs-p14) and then cocultured with HCT8 and A375 cells, which were labeled with mCherry at a ratio of 1:5. The results showed that hMSCs-p14 fused with the neighboring cancer cells and formed multinucleated syncytia; subsequently, the syncytia were shrunk 20 h after coculture and died successively (Fig. 3A and Fig. S7). The process was consistent when mouse MSCs (mMSCs) expressing p14 were cocultured with mouse tumor cells of B16F10 and MC38 (Figs. S8 and S9). After that, the antitumor cell-killing efficacy of hMSCs-p14 was quantified. Human MSCs were transfected with p14-expressing plasmid (hMSCs-p14) and empty plasmid (hMSCs-GFP). Eight hours after transfection, the hMSCs-p14 as effector cells were mixed with the HCT8 or A375 cells at the ratios of 1:5, 1:12.5, and 1:25. Cell viabilities were analyzed after coculturing for 48 h by cell counting kit-8 (CCK8). The data showed that hMSCs-p14 killed tumor cells to different extent. Among this, the killing effect for HCT8 cells at ratios of 1:5, 1:12.5, and 1:25 was 75.5%, 58.6%, and 47.1%, respectively. For A375 cells, the killing efficiency at ratios of 1:5, 1:12.5, and 1:25 was 35.5%, 38.9%, and 22.1% (Fig. 3B and C). In addition, a 48-h real-time cytotoxicity assay (RTCA) was also performed to confirm above findings. hMSCs-p14 were cocultured with cancer cells at a ratio of 1:5 in the xCELLigence RTCA SP system. As shown in Fig. 3D, the cell index curve of HCT8 cells was similar for both groups in the first 3 h. Of note, the slope of the hMSCs-p14 group significantly increased compared with the hMSCs-GFP group at 3 to 15 h. We reasoned that syncytia were formed and they continued to grow in that time, so increased cellular adhesive forces and spreading areas produced higher impedance signals. Subsequently, the impedances were decreased with the syncytia shrinking and detachment. In the end, 56.2% of HCT8 cells were killed at the time point of 48 h. A375 cells have a similar curve, and the killing efficiency was about 70.9% at 48 h (Fig. 3E). These data suggested that MSCs were an ideal vehicle to carry p14 protein that could efficiently fuse with neighboring cancer cells and kill them in vitro.

### MSC carrying p14 inhibited tumor growth in vivo

**Fig. 4.**
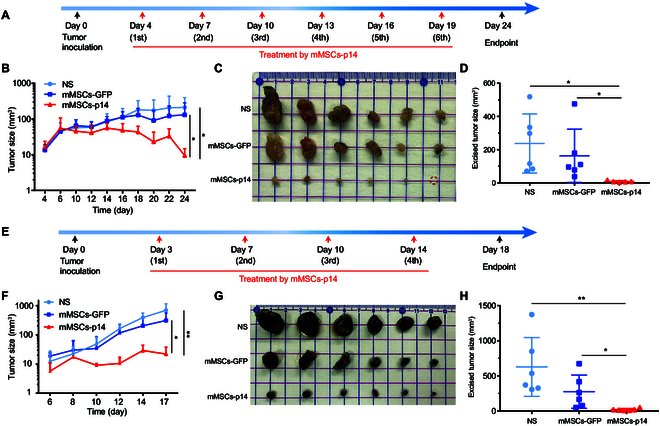
Inhibition of tumor growth by MSCs-p14 in vivo. (A) Schematic of colon cancer tumor model establishment and treatment regimen. Female C57BL/6J mice were injected with 5 × 10^5^ MC38 cells subcutaneously in the right leg. Four days after injection, mice were treated with normal saline, 2 × 10^5^ mMSCs-GFP, and 2 × 10^5^ mMSCs-p14. Mice were treated every 3 d for a total of 6 times. (B) Curves of tumor growth of colon cancer with the treatment of normal saline (NS), mMSCs-GFP, and mMSCs-p14. Tumor sizes were measured every other day and recorded for 24 d. NS versus mMSCs-p14, *P = 0.023; mMSCs-GFP versus mMSCs-p14, *P = 0.047 by one-way ANOVA with Dunnett’s multiple comparisons test (*n* = 6 animals). (C) Photographs and volumes (D) of excised tumor tissue of colon cancer after the last treatment. Data were presented as the mean ± SD. NS versus mMSCs-p14, *P = 0.019; mMSCs-GFP versus mMSCs-p14, *P = 0.042 by one-way ANOVA with Dunnett’s multiple comparisons test (*n* = 6 animals). (E) Schematic of melanoma tumor model establishment and treatment regimen. Female C57BL/6J mice were injected with 5 × 10^5^ B16F10 subcutaneously in the right leg. Three days later, mice were treated with normal saline, 2 × 10^5^ mMSCs-GFP, and equal mMSCs-p14. Mice were treated every 3 d for a total of 4 times. (F) Curves of melanoma tumor growth with the treatment of NS, mMSCs-GFP, and mMSCs-p14. Tumor sizes were measured every other day and recorded for 17 d. NS versus mMSCs-p14, **P = 0.005; mMSCs-GFP versus mMSCs-p14, *P = 0.019. (G) Photographs and volumes (H) of excised melanoma tumor tissue at the end of treatment. Data were presented as mean ± SD. NS versus mMSCs-p14, **P = 0.005; mMSCs-GFP versus mMSCs-p14, *P = 0.025 by one-way ANOVA with Dunnett’s multiple comparisons test (*n* = 6 animals).

Given the above results, we next evaluated the antitumor therapeutic effect of MSCs-p14 in vivo. The mouse models of colon cancer and melanoma were established by subcutaneously injecting 5 × 10^5^ MC38 or B16F10 cells into the right leg of C57BL/6J mice. For the colon cancer model, 4 d after cancer cell implantation, the tumor grew to an average volume of approximately 10 mm^3^. The mice were then randomly assigned into 3 groups of 6 for different treatments: normal saline, 2 × 10^5^ mMSCs-GFP, and 2 × 10^5^ mMSCs-p14. The mice were treated every 3 d for 6 cycles, with tumor growth measured every other day using a vernier caliper (Fig. 4A). There was no significant difference in tumor growth observed between the normal saline group and the mMSCs-GFP group. In contrast, mMSCs-p14 treatment exhibited significant suppressive effect on tumor growth as early as 10 d after treatment and reached a 92.7% inhibition ratio by day 24 (Fig. 4B). At the endpoint, mice were euthanized and the tumors were harvested. The tumor mass sizes in the mMSCs-p14 treatment group were dramatically smaller than the other 2 groups, consistent with the above findings (Fig. 4C and D). For the melanoma model, same grouping and treatment dosages were used with a slightly altered timeline. The treatments started on 3 d after tumor cell inoculation and were administrated every 3 or 4 d for 4 cycles (Fig. 4E). The tumor growth was similar in the normal saline group and the mMSCs-GFP group, whereas mMSCs-p14 treatment significantly inhibited tumor growth as early as 10 d after treatment and reached a 92.9% inhibition ratio by day 18 (Fig. 4F). The sizes of isolated tumor mass in the mMSCs-p14 group were significantly smaller compared to the saline group and the mMSCs-GFP group (Fig. 4G and H). Altogether, these results suggested that MSCs-p14 were able to effectively suppress tumor growth in vivo and have the potential as a novel cytotherapy.

### The activities of natural killer cells and macrophage were enhanced following MSCs-p14 treatment

**Fig. 5.**
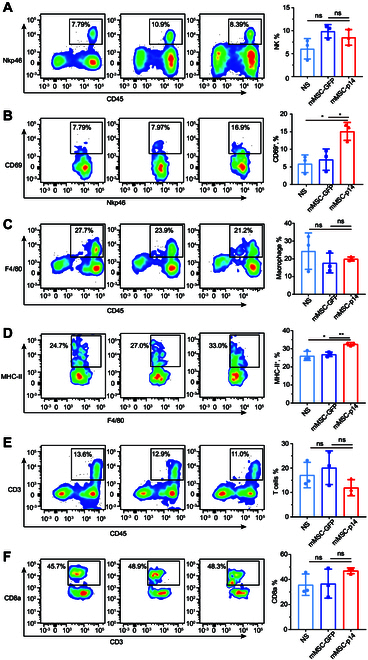
Antitumor immune response was activated following MSCs-p14 therapy. Female C57BL/6J mice were injected subcutaneously with 5 × 10^5^ mouse colon cancer cells of MC38 in the right leg. Subcutaneous tumor was about 200 mm^3^ 10 d after injection. Mice were randomly assigned into 3 groups and treated with normal saline, 2 × 10^5^ mMSCs-GFP, and 2 × 10^5^ mMSCs-p14. Tumor tissues were harvested and isolated into single cell 72 h after treatment. Flow cytometry fluorescence-activated cell sorting (FACS) assessment of the mean fluorescent intensities (MFIs) and percentage of immune cells expressing various cell markers. Percentage positivity of different cell populations was shown. (A) NKp46 expression in immune cells isolated form mouse tumor. Representative flow cytometry plots and quantification analysis of each group (*n* = 3 animals). (B) Cytotoxic marker CD69 expression in NK cells. NS versus mMSCs-p14, *P = 0.011; mMSCs-GFP versus mMSCs-p14, *P = 0.025, by one-way ANOVA with Dunnett’s multiple comparisons test. (C) F4/80 expression in immune cells. (D) Activation marker MHC-II expression in macrophages. NS versus mMSCs-p14, *P = 0.014; mMSCs-GFP versus mMSCs-p14, **P = 0.003, by one-way ANOVA with Dunnett’s multiple comparisons test. (E) CD3 expression in immune cells. (F) CD8a expression in CD3^+^ T cells.

The in vivo antitumor effects observed above were likely primarily due to the lysis of tumor cells induced by mMSCs-p14-mediated cell fusion. Tumor cell lysis was expected to release tumor antigens to enhance the antitumor immune response, particularly with the occurrence of immunogenic cell death during this process. To further investigate if MSCs-p14 treatment could enhance the immune response and contribute to its therapeutic effects, we repeated the in vivo experiment with slight modifications. A more advanced mouse colon cancer model with tumors reaching 200 mm^3^ was used to study the effect of MSCs-p14 on immune activation. mMSCs-GFP (2 × 10^5^), mMSCs-p14 (2 × 10^5^), or normal saline was administrated intratumorally into these mice. Seventy-two hours after treatment, the mice were euthanized, and tumor tissues were harvested for cell isolation. The intratumoral infiltration and activity of natural killer (NK) cells, T cells, and macrophages were analyzed using flow cytometry (Fig. S10). The result showed no significant difference in the intratumoral infiltration of NK cells (Fig. 5A), but the cytotoxic marker CD69 in NK cells in the mMSCs-p14 group was significantly increased (Fig. 5B). We also evaluated the intratumoral infiltration of macrophages and found that there was no significant change among 3 treatments, whereas the activation marker of MHC-II (major histocompatibility complex II) in macrophages was significantly increased after mMSCs-p14 treatment (Fig. 5C and D). There was no notable difference in the intratumoral infiltration of T cells, or in the proportion of CD8-positive T cells, among 3 groups (Fig. 5E and F). The results suggested that the enhanced activity of NK cells and macrophages may also contribute to the therapeutic effect of MSCs-p14.

### Safety improvement of MSCs-p14 by introducing doxycycline-controlled transcriptional system to regulate cell–cell fusion

**Fig. 6.**
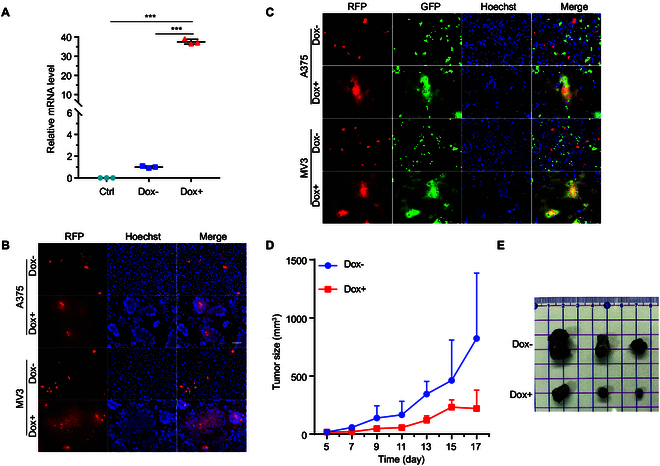
Dox-induced transcriptional system improved safety of MSCs-p14. p14 gene was inserted into pCW57 vector with 7× tet operator and expressing rTetR protein. (A) Plasmid of pCW57-p14 was transfected into A375 cells, and 2.5 μg/ml Dox was added to induce p14 expression 24 h after transfection. p14 mRNA level was analyzed by qPCR. Data were presented as the mean ± SD. ***P < 0.001 by one-way ANOVA with Dunnett’s multiple comparisons test (*n* = 3 technical replications). (B) Fluorescence images showed Dox initiating p14 protein expression and subsequently inducing cell fusion. (C) Images of hMSCs-tet-p14 fused with cancer cells. Plasmid of pCW57-p14 was transfected into hMSCs and mixed with A375 and MV3 cells (expressing GFP). Dox was added and reacted for another 20 h. Nuclei were stained with Hoechst 33342. Scale bar, 100 μm. (D) Characterization of mMSCs-tet-p14 inhibiting tumor growth in vivo. Female C57BL/6J mice were injected with 5 × 10^5^ B16F10 subcutaneously in the right leg. Five days after inoculation, mice were randomly assigned into 2 groups and treated intratumorally with 2 × 10^5^ of mMSCs-tet-p14 and intraperitoneal injection of phosphate-buffered saline or 40 μg of Dox to activate p14 expression in the next day. Mice were treated every 4 d for a total of 3 times. Tumor sizes were measured every other day and recorded for 17 d. (E) Image of isolated tumor tissue at the end of treatment.

To improve the safety of MSCs-p14 therapy, the tetracycline-controlled transactivator (Tet-On) system was introduced to regulate the expression of p14, which initiates the cell–cell fusion. Briefly, the pCW57-p14 plasmid, containing a p14 coding gene driven by a Tet-On promoter, was constructed and used for transfecting MSCs, establishing MSCs-tet-p14. In the presence of 2.5 μg/ml doxycycline (Dox), the transcription level of p14 increased 37-fold (Fig. 6A). When hMSCs-tet-p14 were added into A375 or MV3 tumor cells, cell–cell fusion and syncytia were only observed in the Dox-induced group but not in the Dox-negative group (Fig. 6B and C). To evaluate the therapeutic effect of MSCs-tet-p14, a subcutaneous melanoma tumor model was established by injecting 5 × 10^5^ B16F10 cells in C57BL/6J mice. Five days after tumor implantation, the mice were intratumorally injected with 2 × 10^5^ mMSCs-tet-p14 and then randomly assigned into 2 groups. One was intraperitoneally injected with 40 μg/mice Dox after 24 h, and the control group was injected with phosphate-buffered saline (PBS). Mice were treated every 4 d for 3 cycles, and tumor growth was measured every other day with vernier caliper. Dox-induced mMSCs-tet-p14 treatment exhibited significant suppressive effect on tumor growth as early as 9 d after treatment and reached a 73.3% inhibition ratio by day 17. The data showed that Dox-induced mMSCs-tet-p14 treatment was able to inhibit tumor growth (Fig. 6D and E). We have essentially achieved a controllable modification of MSC-p14 therapy while ensuring its therapeutic efficacy.

## Discussion

In our previous study, we found that enhancing the syncytial characteristic of oncolytic herpes viruses to induce stronger cell–cell fusion significantly improved the intratumoral viral spread and antitumor efficacy of oncolytic viruses [17]. It piqued our curiosity about the extent to which cell fusion contributed to the antitumor effect in these processes. Therefore, in this study, we first explored using a viral fusogenic protein p14 to induce cell fusion among tumor cells and tested its antitumor effect. The results demonstrated that p14 transfection efficiently induced cell–cell fusion and syncytium formation among colon cancer and melanoma cells, rapidly leading to extensive cell lysis and death. The syncytia appeared at 8 h after transfection and continued to fuse with neighboring cells over time. Syncytia contained about 21 nuclei in HCT8 and 19 nuclei in A375 at 16 h. Almost all cancer cells died within 24 h after transfection. These findings indicated a direct and potent cell-killing effect of cell–cell fusion, which was sufficient to kill cancer cells, even without the cytopathic effect of the oncolytic virus. These findings underscored the potential of inducing cell fusion as an innovative cell-killing mechanism for developing antitumor biotherapies.

Given that oncolytic viruses are easily cleared by the immune system resulting in a short duration of action [24,25], they might not be an ideal vehicle to fully exploit the cell-killing effect of cell fusion. Wong et al. [18] demonstrated that incorporating the p14 protein into oncolytic adenoviruses let to significant improvements in virus replication and spreading in vitro. However, no significant therapeutic advantages were observed in vivo. Compared to oncolytic viruses, engineered MSCs appear to be a better choice for delivering viral fusogenic proteins to treat cancers because of their lower immunogenicity, which allows for longer persistence in action. Their lower toxicity and tumor-homing characteristics make them more feasible for achieving intravenous administration [26]. Therefore, we next construed the engineered MSCs carrying a coding gene of p14 protein (MSCs-p14) and tested their antitumor therapeutic effect in vitro and in vivo. The results showed that MSCs-p14 induced cell fusion and caused cancer cell death efficiently in vitro and also significantly suppressed the tumor growth in vivo. Although specific mechanism needs to be further explored, MSCs carrying p14 definitely inhibited the growth of solid tumors in our work.

Next, we studied the mechanism by which MSCs-p14 exerts its antitumor efficacy. Regarding syncytia death, we observed chromatin condensation and marginalization in syncytial nuclei, along with caspase-3/7 activation in the syncytial cytoplasm. Furthermore, RNA-seq and quantitative analysis demonstrated that the mRNA levels of apoptosis-related genes were up-regulated at later stages of transfection. This suggests that the p14 protein induced syncytial cell death in a manner characteristic of apoptosis, consistent with previous work indicating that FAST proteins induce extensive formation and trigger apoptosis to disrupt membrane stability, leading to syncytial cell death [27,28]. Additionally, we observed pyroptosis vesicles adhering to the crumpled syncytia and detected cleaved GSDME protein. These findings demonstrated that MSCs-p14 could directly induce tumor syncytium cell death by activating apoptosis and pyroptosis signaling. Furthermore, the immunogenic programmed death of tumor syncytia may further activate the body’s immune response, thereby enhancing its antitumor effect [29]. We next studied the changes in immune cells within tumors during MSCs-p14 therapy. The flow cytometry results demonstrated that although the cell numbers did not show a clear change, the activity of NK cells and macrophages was increased in tumor, indicating that NK cells and macrophages enhanced the therapeutic effect of MSCs-p14.

Our research aims to develop a biotherapy for treating solid tumors by inducing tumor cell fusion. The current study represents an initial exploration in this direction. While it demonstrated some antitumor efficacy in mouse models, several unresolved issues remain. For instance, MSCs-p14 developed in this study lacks specificity for targeting tumors when inducing cell fusion, potentially posing safety risks. In our upcoming research, we will focus on achieving selective induction of tumor-targeted cell fusion by switching to other viral fusogenic proteins and employing tumor-targeting modifications. Another challenge is achieving precise control over cell fusion to enhance therapy safety [30,31]. In this study, we introduced the Tet-On system to regulate the expression of the p14 gene (tet-p14) constructed MSCs-tet-p14, which expressed p14 only when Dox was present. Another concern involves MSCs, whose role in tumor progression was controversial [32]. Some reports suggested that MSCs may promote tumor growth and progression through mechanisms such as immunosuppression, angiogenesis promotion, and enhancement of metastasis [33,34]. However, other studies indicated that MSCs could also exhibit antitumor effects by suppressing tumor growth and modulating the immune response [35,36]. The overall outcome of MSC treatment may depend on the specific tumor environment [37]. In our study, treatment with mMSCs-GFP in the mouse tumor models appeared to slightly suppress tumor growth; however, the effect was not statistically significant. Although we did not observe MSCs promoting tumor progression, it is still necessary to consider selecting a better cell vehicle for this therapy.

In conclusion, by incorporating the FAST protein p14 from reovirus into cancer cells, we identified that inducing cell–cell fusion led to cell death through apoptosis and pyroptosis, demonstrating that cell fusion can be used as a novel cell-killing mechanism for developing antitumor therapy. Furthermore, when MSCs-p14, which carry the coding gene for the p14 protein, were cocultured with tumor cells, they initiated cell fusion and caused widespread cancer cell death in vitro. In subcutaneous tumor models, MSCs-p14 also showed significant therapeutic effects, significantly suppressing tumor growth. Taken together, our results demonstrated that using engineered cells to carry viral fusogenic proteins is a potential way to develop novel antitumor biotherapies, which hold great promise for improving the treatment of solid tumors.

## Materials and Methods

### Cell culture

Human colon cancer cell lines HCT8, LOVO, SW480 and the mouse colon cancer cell line MC38 were obtained from Gastroenterology of Affiliated Hospital of Xuzhou Medical University. Three human melanoma cell lines (A375, MV3, and M14) and one mouse melanoma cell line (B16F10) were obtained from the Department of Immunology, Xuzhou Medical University. Human cord blood MSC (hMSC) was obtained from Jiangsu Institute of Cancer Biotherapy. mMSC was purchased from Culture Collection of Tongpai Biotechnology Co. Ltd. (Shanghai, China). Cancer cell lines were cultured in Dulbecco’s modified Eagle’s medium (DMEM) with 10% fetal bovine serum (FBS; ExCell Bio) and 100 units/ml penicillin–streptomycin (Gibco). hMSC was grown in DMEM/F-12K (1:1), and mMSC was cultured in DMEM supplemented with 10% FBS (Gibco). All cells were incubated at 37 °C with 5% CO_2_ and 95% humidity.

### Plasmid transfection and cell staining

p14 gene sequence and plasmids of PCDH-GFP and PCDH-mCherry were synthesized in Genscript Co. Ltd. Plasmids of pCW57-RFP were purchased from Miaolingbio Co. Ltd., and pCW57 (#71782) was obtained from University of Macau. PCDH-p14, PCDH-mCherry-p14, and pCW57-p14 were constructed in our laboratory and sequenced by Sangon Biotech.

Cancer cells were transfected with appropriate plasmids with Lipofectamine 2000 (Invitrogen, USA) according to the manufacturer’s instruction. In brief, 1.5 × 10^5^ cells were seeded in 12-well plates and cultured overnight. Plasmids were diluted in OPTI-MEM and transfected into cells when the cell density was about 70% to 80%, subsequently changing fresh medium after incubating for 5 h. Plasmids were transfected into hMSC and mMSC with jetOPTIMUS (Polyplus) according to protocol. Briefly, plasmids were diluted in jetOPTIMUS buffer first and shaken for 1 s, and then jetOPTIMUS reagent was added into the DNA buffer solution and incubated at room temperature for 10 min. The mixture was added into cells and incubated for 8 h.

In the cell fusion induced by transfection with p14 plasmid, cell–cell fusion was assayed 16 h after transfection. Cell membrane was labeled with 5 μg/ml CellMask (Invitrogen, USA) at 37 °C for 10 min. Cell nuclei were stained with 1× Hoechst 33342 (Beyotime, China) at 37 °C for 15 min. After staining, cells were washed with PBS for 3 times, 5 min each time. Cells were fixed with 4% paraformaldehyde and imaged with a fluorescence microscope (Olympus, Japan) using 10× objective.

In the assay of p14-positive cells fused with p14-negative cells, cells were transfected with PCDH-GFP-p14 or PCDH-mCherry. Two cells were digested and mixed 5 h after transfection. Cell–cell fusion was analyzed after incubation for 16 h. Nuclei were stained with 1× Hoechst 33342 at 37 °C for 15 min. Finally, cells were imaged on a fluorescence microscope after 4% paraformaldehyde fixation.

### Immunoblot analysis

Western blot was performed as previously described. The cell samples were collected 20 h after transfection and analyzed with 12% SDS-PAGE (polyacrylamide gel electrophoresis). Anti-PRAP1 (Proteintech, 11932-1-AP), anti-GSDME (Abcam, ab222408), anti-β-actin (Absin, abs830031), and DyLight-modified anti-rabbit IgG (H+L) (Cell Signaling Technology, 5151) were used.

### RNA-seq and bioinformatic analysis

Human colon cancer cells of HCT8 were transfected with PCDH-GFP or PCDH-GFP-p14. Eighteen hours after transfection, 1 × 10^7^ cells were harvested with TRIzol Reagent (Ambion, USA) and collected to ribonuclease (RNase)-free tubes. RNA sequencing was performed at Gene Denovo Biotechnology Co. (Guangzhou, China). Total RNA was extracted using TRIzol reagent kit (Invitrogen, USA) according to the manufacturer’s protocol. RNA quality was assessed on an Agilent 2100 Bioanalyzer (Agilent Technologies, USA) and checked using RNase-free agarose gel electrophoresis. After total RNA extraction, mRNA was enriched by oligo(dT) beads. The enriched mRNA was fragmented into short fragments using fragmentation buffer and reversely transcribed into cDNA by using NEBNext Ultra RNA Library Prep Kit for Illumina (#7530, New England Biolabs, USA). The purified double-stranded cDNA fragments were end repaired, A base added, and ligated to Illumina sequencing adapters. The ligation reaction was purified with the AMPure XP beads (1×). Ligated fragments were subjected to size selection by agarose gel electrophoresis and PCR amplification. The resulting cDNA library was sequenced using Illumina Novaseq6000.

During the sequencing process, reads obtained from the sequencing machines were further filtered by fastp (version 0.18.0). An index of the reference genome was built, and paired-end clean reads were mapped to the reference genome using HISAT2. 2.4. The mapped reads of each sample were assembled by using StringTie v1.3.1 in a reference-based approach. For each transcription region, a FPKM (fragments per kilobase million) value was calculated to quantify its expression abundance and variations using RSEM software. RNA differential expression analysis was performed by DESeq2 software between 2 different groups. The genes/transcripts with the parameter of FDR below 0.05 and absolute fold change ≥2 were considered DEGs/transcripts. Software GSEA and MSigDB were used for GSEA, and pathway enrichment analysis was identified by KEGG (Kyoto Encyclopedia of Genes and Genomes).

### Real-time quantitative PCR

Cells were transfected with PCDH-GFP or PCDH-GFP-p14 plasmids, and the lysate was collected at 18 h after transfection. Total RNA was extracted using FastPure Cell/Tissue Total RNA Isolation Kit V2 (#RC112-01, Vazyme, China) as described in the manufacture’s protocol, and RNA quality was detected by NanoDrop2000 (Thermo Fisher Scientific, USA). The RNA was reverse-transcribed into cDNA using HiScript II Q RT SuperMix for quantitative PCR (qPCR) (+gRNA wiper) (#R223-01, Vazyme). The reverse transcription was performed in a 20-μl reaction containing 1 μg of total RNA, 4× gDNA wiper mix, and 5× HiScript III RT SuperMix. Later, cDNA was subjected to qPCR analysis using ChamQ SYBR qPCR Master Mix (#Q331-02, Vazyme). The real-time qPCR was performed in a 20-μl reaction containing 1 μl of cDNA template, 0.5 μM primers, and 2× master mix (Taq DNA polymerase, deoxynucleotide triphosphates, and reaction buffer). The reaction was initiated on a LightCycler 480 II instrument (Roche, Switzerland). Glyceraldehyde-3-phosphate dehydrogenase (GAPDH) was used as an internal control, and PCR products were evaluated by analyzing melting curves. qPCR amplification included preincubation at 95 °C for 2 min, followed by 40 cycles at 95 °C for 15 s, 58 °C for 30 s, and 72 °C for 30 s. Primers were listed in Table S1. Relative mRNA level was shown as fold change following the 2^−ΔΔCt^ calculation method. In the experiment of Dox-controlled p14 expression, the cells were transfected with pCW57-p14, adding tetracycline to induce p14 expression 24 h after transfection, and the cell lysate was collected for another 20 h. The subsequent procedures were consistent to described above.

### Nuclei count

HCT8 and A375 cells were transfected with PCDH-GFP and PCDH-GFP-p14 plasmids with Lipofectamine 2000. Sixteen hours after transfection, cell nuclei were stained with Hoechst 33342. Twenty fields of view were collected from the transfected cells with a fluorescence microscope using the 20× objective. Cells with more than 2 nuclei were considered multinucleated syncytia, and nuclei per syncytia were counted manually.

### Caspase-3/7 staining

Cells were transfected with PCDH-mCherry or PCDH-mCherry-p14 plasmids with Lipofectamine 2000. Eighteen hours after transfection, cells were stained with Caspase-3/7 Green Detection Reagent (#C10723, Invitrogen). Briefly, the Caspase-3/7 reagent was diluted in PBS with 5% FBS and incubated with cells at 37 °C for 30 min. Cell nuclei were stained with Hoechst33342 after washing 3 times with PBS. Finally, cells were fixed with 4% paraformaldehyde and imaged with a fluorescence microscope.

### Enzyme-linked immunosorbent assay

Levels of human IL-18 and IL-1β were measured in the cell supernatants of A375 and HCT8 using ELISA kits from Proteintech (KE00193 and KE00021, respectively). ELISAs were performed following the manufacturer’s instructions. Cell culture supernatants (100 μl) were used and analyzed on the Cytation2 imaging reader instrument (BioTek).

### Cell viability assay

To evaluate the killing effect of MSCs-p14 on cancer cells, plasmids of PCDH-GFP or PCDH-GFP-p14 were transfected into hMSCs and cocultured with cancer cells in ratios of 1:5, 1:12.5, and 1:25 at 8 h after transfection. Cell viability was analyzed by CCK8 48 h after coculture. In addition, real-time recording was performed using xCELLigence RTCA SP system (Agilent, USA). To ensure data accuracy, 50 μl of DMEM was added to each well of the E-plate 96. The plate was subsequently incubated at 37 °C for 30 min to minimize any potential effects of the medium. Then, hMSCs transfected with PCDH-GFP or PCDH-GFP-p14 were mixed with HCT8 and A375 cells in a ratio of 1:5. The cells were monitored every 5 min to obtain the cell index for 48 h.

### In vivo therapeutic experiments

All animal care and handling procedures were approved by the Ethics Committee of Xuzhou Medical University and were performed in accordance with National Institutes of Health (NIH) guidelines. Female C57BL/6J mice (6 to 8 weeks old, average 20-g body weight) were purchased from GemPharmatech Co. Ltd. Mice were maintained on a 12-h light/dark cycle with free access to food and water. Mouse colon cancer cell MC38 and melanoma cell B16F10 were prepared, and concentrations were adjusted to 5 × 10^6^ cells/ml in normal saline. Mice were injected with 5 × 10^5^ cancer cells subcutaneously in the right leg to establish transplanted tumor models. In the colon cancer model, when the tumor sizes reached 10 mm^3^ on day 4 after the tumor implantation, mice were assigned into 3 groups and intratumorally injected with normal saline, 2 × 10^5^ mMSCs-GFP, and equal mMSCs-p14. Mice were treated every 3 d for a total of 6 times. Tumor sizes were measured every other day. At the end of experiment, the tumors were isolated and imaged. In the melanoma model, the treatment was performed near the tumor injection position on day 3 after the tumor implantation. Mice were treated every 3 or 4 d for 4 times. Tumor sizes were measured from 6 d after implantation and recorded every other day. At the end of the experiment, the tumors were isolated and imaged.

### Immune cell assay by flow cytometry

For ex vivo flow cytometric assessment of murine immune cells, the mice were injected subcutaneously with 5 × 10^5^ MC38 cells into the right leg of C57BL/6J mice. Then, the tumors reaching 200 mm^3^ were used to study the effect of MSCs-p14 on immune activation. mMSCs-GFP (2 × 10^5^), mMSCs-p14 (2 × 10^5^), or normal saline was administrated intratumorally into these mice. The mice were euthanized after 72 h of mMSCs-p14 treatment. Subcutaneous tumors were harvested and isolated into single cells. The cells were stained with anti-CD45 (BD, 103133), anti-NKp46 (BD, 137603), anti-CD69 (BD, 104513), anti-F4/80 (BD, 123107), anti-MHC-II (major histocompatibility complex II)(BD, 107625), anti-CD3 (BD, 100235), and anti-CD8a (BD, 100705) antibodies for flow cytometric analysis of immune cells. The assessments of immune cells were performed with at least 3 independent animals. All flow cytometry data were collected using a Fortessa cytometer (BD Biosciences). The flow cytometry gating strategy is described in Fig. S9.

### Tetracycline-controlled transactivator (Tet-On) system regulated p14 expression

Melanoma cells of A375 and MV3 were transfected with pCW57-p14 using Lipofectamine 2000. Twenty-four hours after transfection, 2.5 μg/ml Dox was used to induce p14 expression. Cells were imaged with a fluorescence microscope after incubating at 37 °C for 16 h, and nuclei were stained with Hoechst 33342. Human MSCs were transfected with pCW57-p14 with jetOPTIMUS reagent. Eight hours after transfection, human MSCs-tet-p14 were digested and mixed with melanoma cells A375 and MV3 expressed with GFP protein, respectively. Cells were cocultured for another 16 h. Finally, the syncytia were photographed with a fluorescence microscope.

For in vivo experiment, mice were injected subcutaneously with 5 × 10^5^ B16F10 in the right leg to establish transplanted tumor models. When the tumors reached 10 mm^3^ on day 5 after the tumor implantation, mice were intratumorally injected with 2 × 10^5^ mMSCs-tet-p14. Mice were assigned into 2 groups 24 h after injection for treatments with 40 μg of Dox (2 mg/kg body weight) and the same volume of normal saline by intraperitoneal injection. The treatment was performed every 4 d for a total of 3 times, and tumor sizes were recorded every other day. At the end of experiment, the tumors were isolated and imaged.

### Statistical analysis

All of the statistical and graphical analysis was performed by Prism 7.04. Sample comparisons were performed using multiple *t* test adjusted by FDR or one-way analysis of variance (ANOVA) with Dunnett’s multiple comparisons test. Each experiment was repeated independently at least 3 times.

## Ethical Approval

The study was approved by the Ethics Committee for Experimental Animals of Xuzhou Medical University (ethics approval number: 202112A230). All animal care and experimental procedures complied with the WMA (the World Medical Association) statement on animal use in biomedical research.

## Data Availability

The data supporting the conclusions of this study are available from the corresponding author upon reasonable request.
