# Supplementary material for: Mesenchymal Stem Cells Carrying Viral Fusogenic Protein p14 to Treat Solid Tumors by Inducing Cell–Cell Fusion and Immune Activation
Source: Research (Wash D C). 2025 Jan 27;8:0594. doi: 10.34133/research.0594 (PMC11770199; doi:10.34133/research.0594)
Supplement: Supplementary 1 — Figs. S1 to S10 Table S1 Movies S1 and S2 [file research.0594.f1.zip › Res supplementary_revised.docx]

**Supplementary Information for**

**Mesenchymal stem cells carrying viral fusogenic protein to treat solid tumors by inducing cell-cell fusion and immune activation**

Yao Wang^1,2,3,#^, Xunlei Pang^1,4,#^, Ruirui Li^1,2,3#^, Jiuzhou Chen^5^, Chen Wen^1,2,3^, Huihuang Zhu^1,2,3^, Tingyu Long^1,2,3^, Jianjie Li^1,2,3^, Lijun Zheng^1,2,3^, Youcai Deng^6,^*, Junnian Zheng^2,3,^* and Bo Xu^1,2,3,^*

*Corresponding author: Youcai Deng, Junnian Zheng & Bo Xu

*Correspondence: youcai.deng@tmmu.edu.cn, jnzheng@xzhmu.edu.cn, xubo@xzhmu.edu.cn

This PDF file includes:

Supplementary Figures 1 to 10

Supplementary Table 1


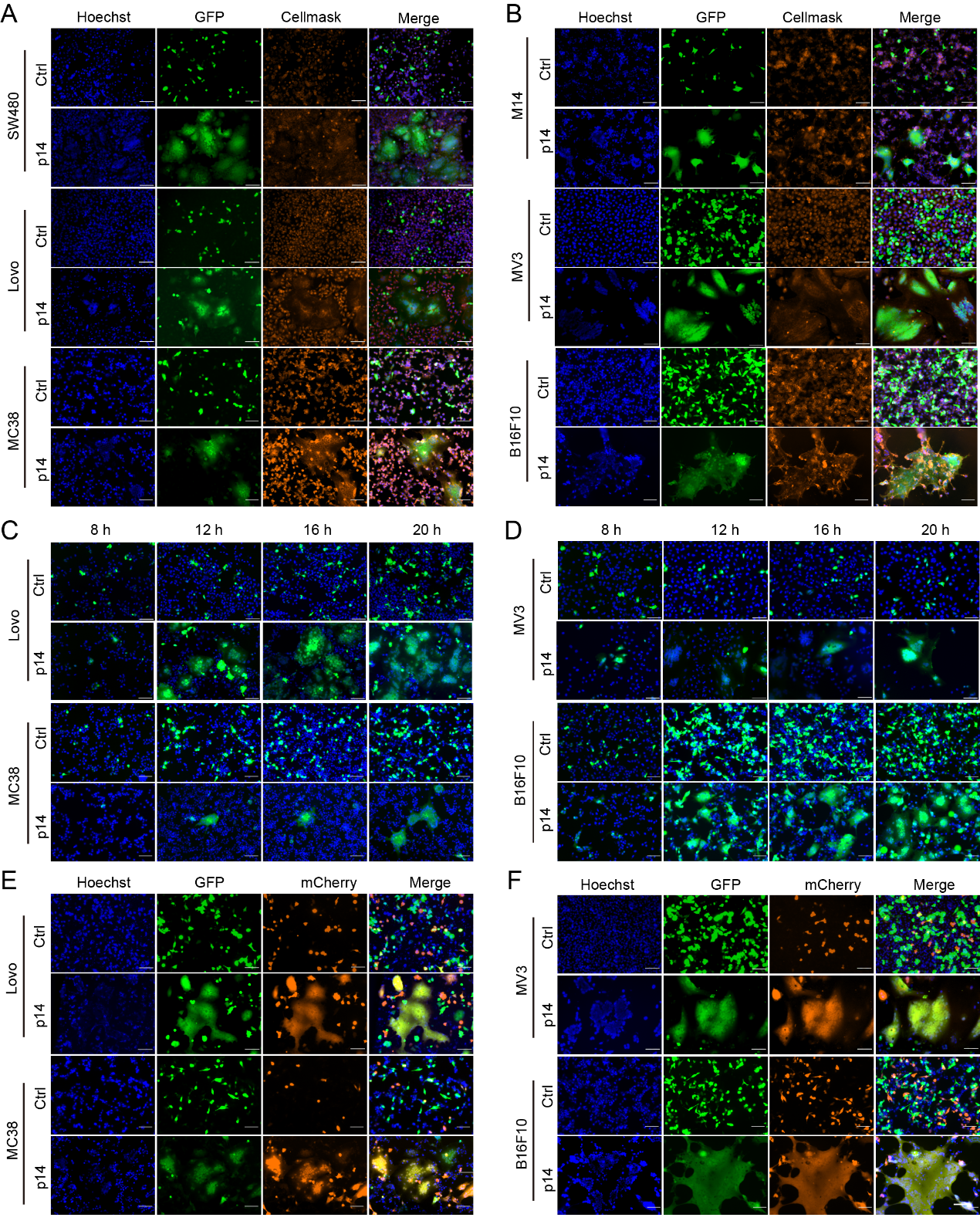


**Suppl. Figure 1. P14 protein expression induced extensive cell-cell fusion of cancer cells. (A)** Image of colon cancer cells and melanoma cells **(B)** fusion triggered by p14 protein expression. Cells were transfected with PCDH-GFP or PCDH-GFP-p14 plasmids. Sixteen hours after transfection, cell nuclei and membrane were stained with Hoechst 33342 and Cellmask, respectively. Scale bar was 100 μm. **(C) and (D)** Time-lapse images of syncytia formation in colon cancer cells of Love and MC38 cells (C) or melanoma cells of MV3 and B16F10 cells. The fused cells induced by p14 protein continuously fused with peripheral and distant cells. Syncytia structures were shown at 8, 12, 16 and 20 hours. The nuclei were stained with Hoechst33342. Scale bar: 100 μm. **(E) and (F)** P14-positive cells fused with adjacent p14-negative cells. Colon cancer cells of Lovo and MC38 (E) or melanoma cells of MV3 and B16F10 (F) were transfected with plasmids of PCDH-GFP or PCDH-GFP-p14. The cells were digested 6 hours after transfection, as effector cells, mixed with appropriate cancer cells expressed mCherry protein, and cocultured for further 20 hours. The nuclei were stained with Hoechst 33342 and scale bar was 100 μm.


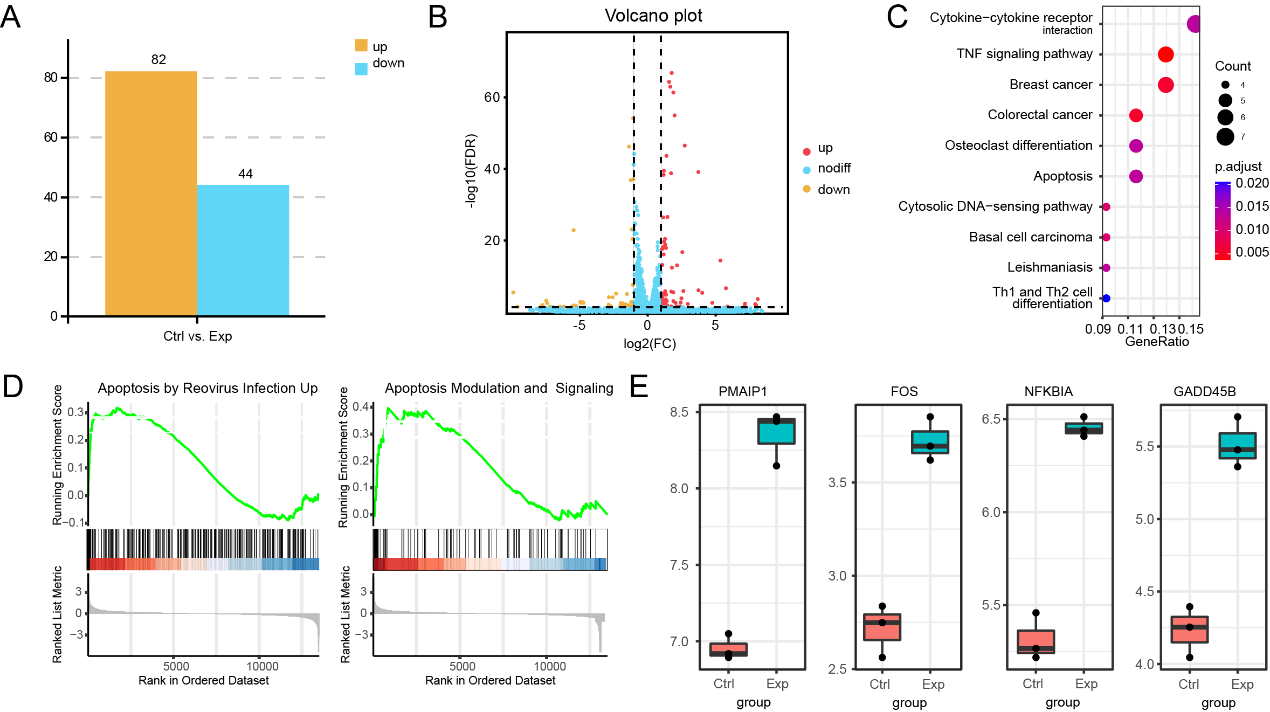


**Suppl. Figure 2** **P14 protein-induced transcriptome changes suggested the activation of apoptosis-related pathway in cell fusion.** In RNA-sequencing, HCT8 cells transfected with plasmids of PCDH-GFP or PCDH-GFP-p14 were used as sample cells. Ten million of cells were collected for transcriptome sequencing. **(A)** A total of 126 genes were differentially expressed in p14-expressed group compared to control group. **(B)** Volcano plot of differentially expressed genes. Red plots represent FDR < 0.05, log2FoldChange > 1 and yellow plots represent FDR < 0.05, log2FoldChange < -1. **(C)** Bubble map of pathway analysis for the DEGs. X-axis: the enrichment ratio of DEGs. Y-axis: the name of enriched pathways. The size of the node represented the number of enriched differential genes. The p value was represented by a color scale, suggesting statistical significance**. (D)** Results of GSEA suggested activation of apoptosis-related pathway. **(E)** Transcriptional signal levels of apoptosis-related genes in p14 transfection group were upregulated compared to control group.


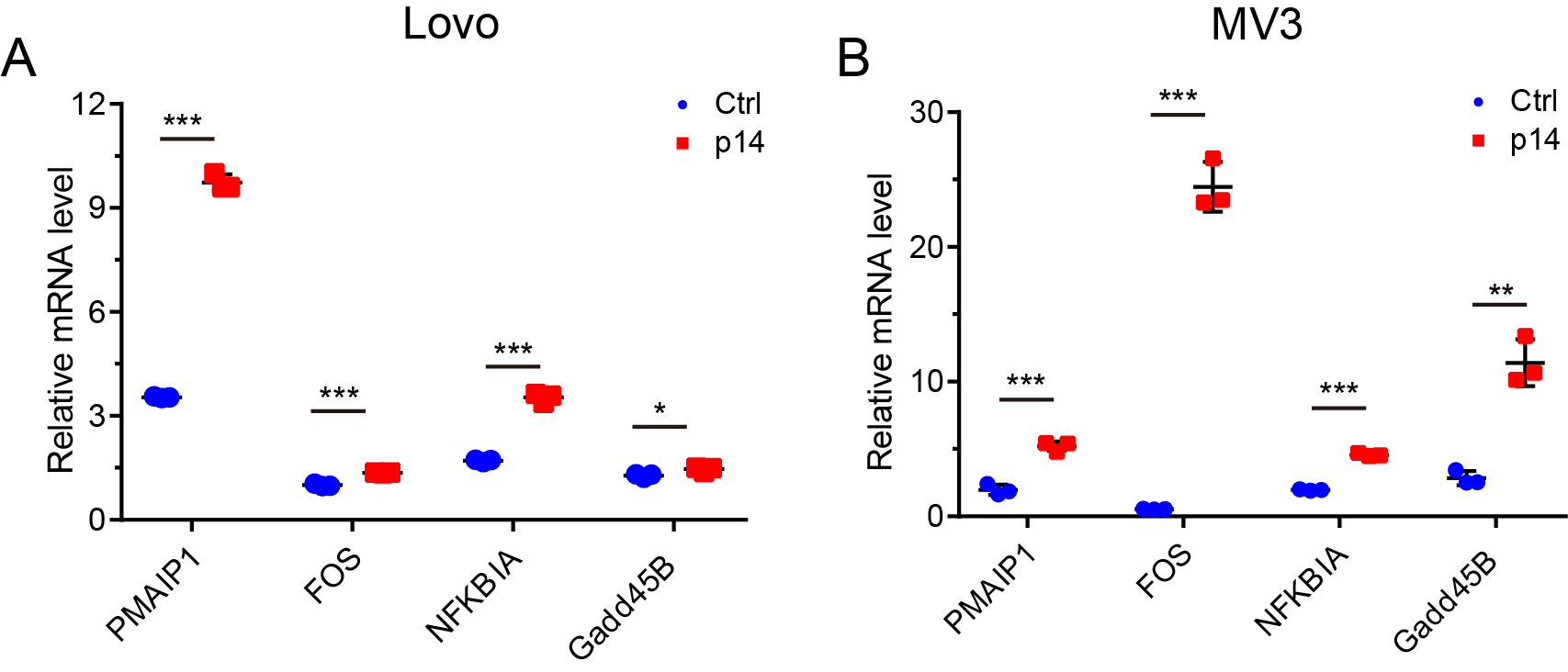


**Suppl. Figure 3 Upregulation of apoptosis related genes mRNA in cell-cell fusion.** Plasmids of PCDH-GFP or PCDH-GFP-p14 were transfected into Lovo and MV3 cells. Messenger RNA levels of apoptosis related genes were analyzed by real-time quantitative PCR. Data were presented as mean ± s.d. (n = 3 technical replicates). Samples comparisons were performed using multiple t test corrected by the false discovery rate. **(A)** In Lovo cells, Ctrl vs. p14 in PMAIP1 *** *P* < 0.001; in FOS *** *P* < 0.001; in NFKBIA *** *P* < 0.001; Gadd45B * *P* = 0.0192. **(B)** In MV3 cells, Ctrl vs. p14 in PMAIP1 *** *P* < 0.001; in FOS *** *P* < 0.001; In NFKBIA *** *P* < 0.001; in Gadd45B ** *P* = 0.001.


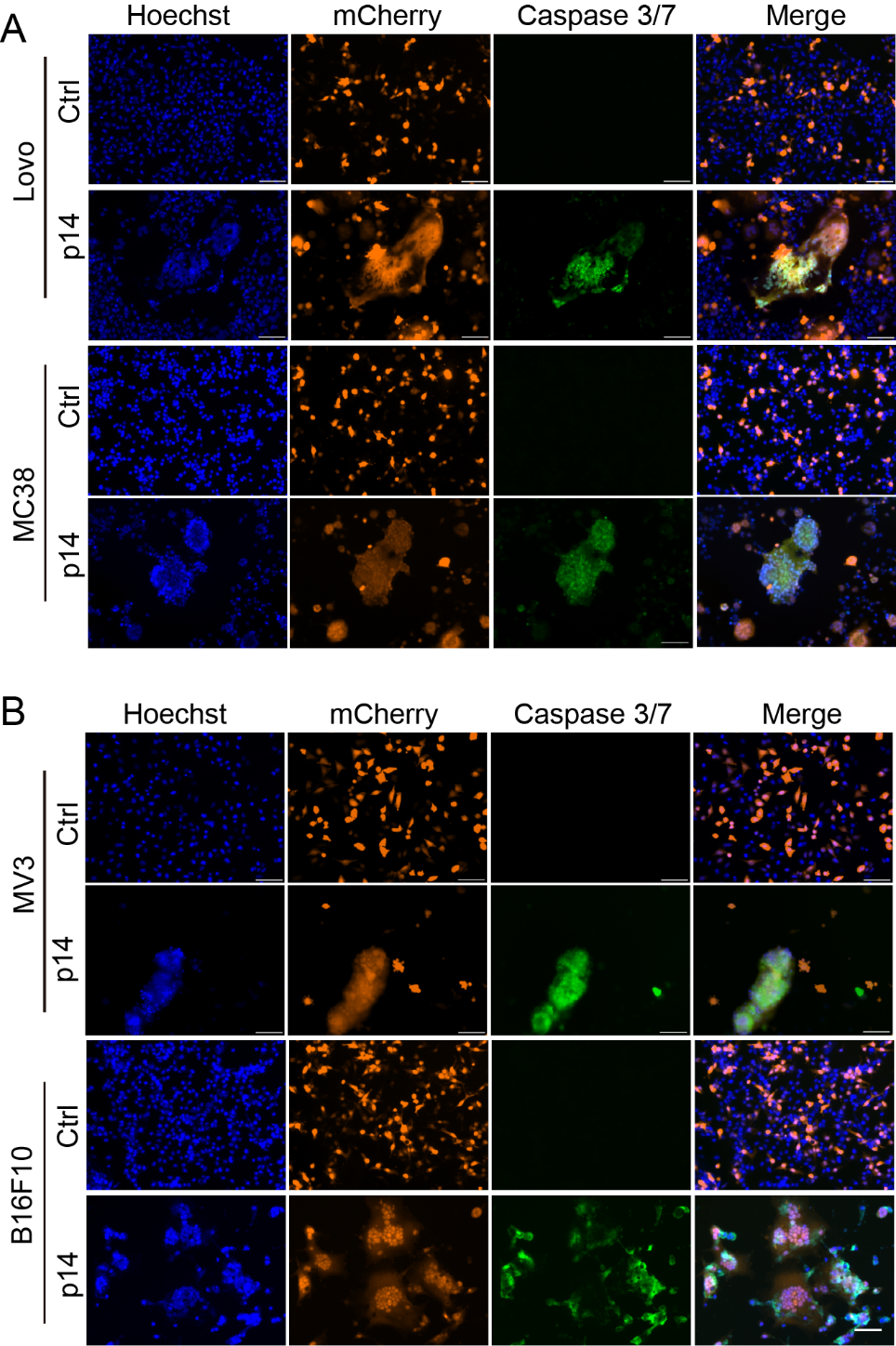


**Suppl. Figure 4.** **Apoptosis was activated in the late stage of cell fusion**. Plasmids of PCDH-mCherry and PCDH-mCherry-p14 were transfected into cancer cells. Shrunken syncytia were formed 20 hours after transfection and marked with caspase3/7 probe. Nuclei were stained with Hoechst 33342. Scale bar was 100 μm.


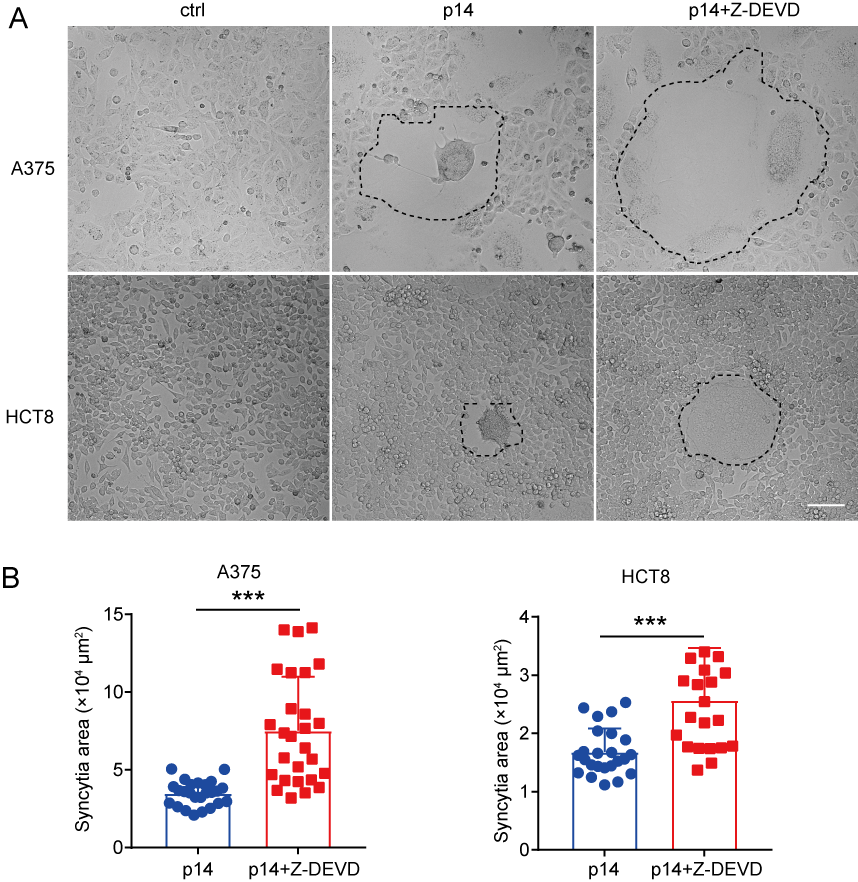


**Suppl. Figure 5. Inhibition of pyroptosis slows the shrinkage and lysis of p14-induced syncytia.** **(A)** The cells were transfected with plasmids of PCDH-GFP or PCDH-GFP-p14 and fresh medium was changed three hours after transfection, 20 μM Z-DEVD was added in one of p14-transfected cells and incubated for another 24 hours. The images were collected by ZEISS microscope. The black dotted line outlines the area of syncytia, scale bar was 100 μm. **(B)** Effect of pyroptosis inhibitor on syncytial elongation and death was evaluated by randomly calculating the area of syncytial in A375 and HCT8 cells treated with or without Z-DEVD after p14 transfection. Samples comparisons were performed using multiple t test corrected by the false discovery rate, p14 vs p14 + Z-DEVD *** *P* < 0.001.


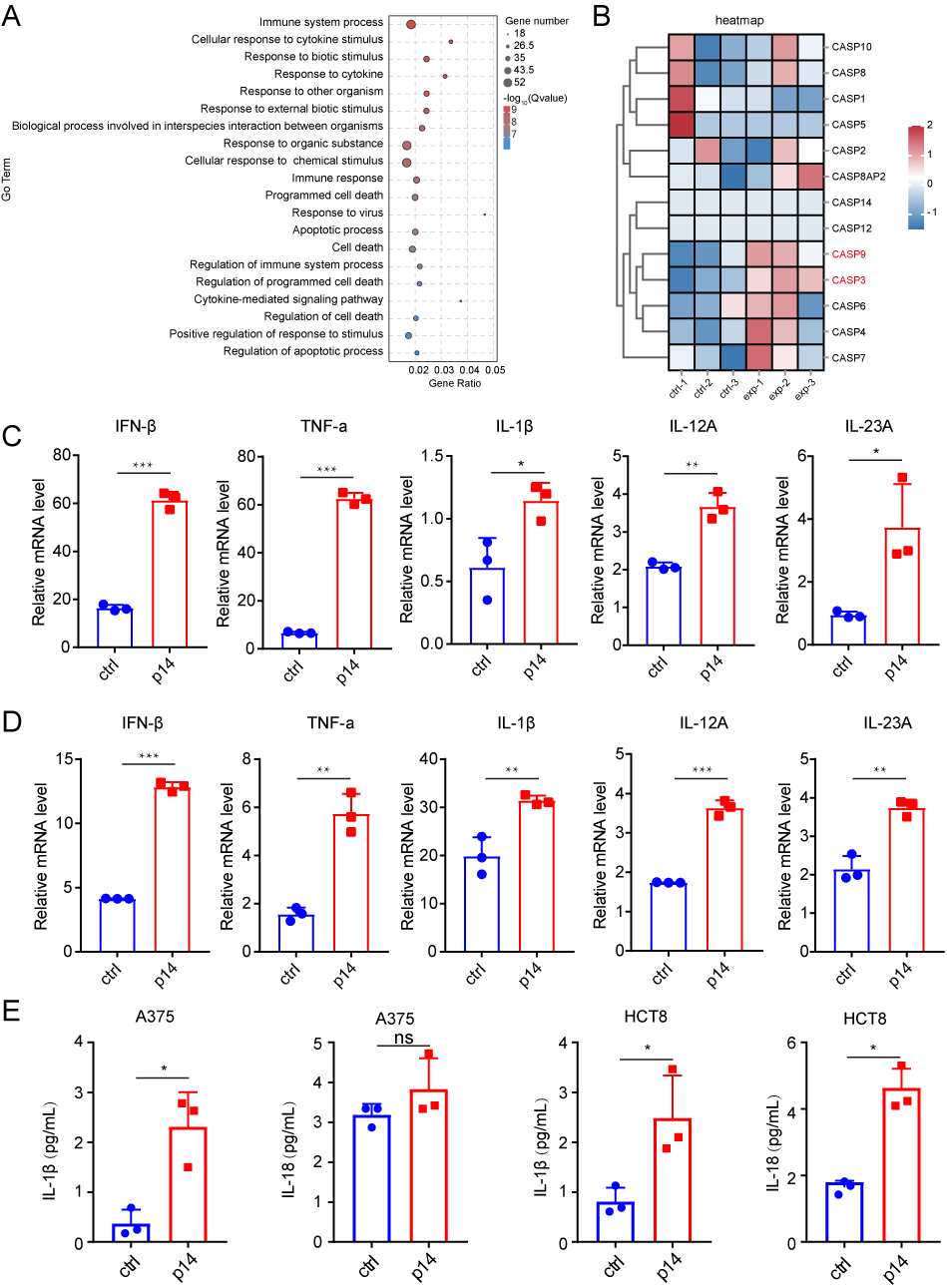


**Suppl. Figure 6. Inflammatory responses are increased in p14-induced syncytia. (A)** GO Enrichment Analysis on RNA-Seq Data. **(B)** Heatmap showed changes of caspase family membranes. **(C)** and **(D)** The transcription level of IFN-β, TNF-α, IL-1β, IL-12A and IL-23A was significantly increased after cell-cell fusion in A375 and HCT8 cells. Data were presented as mean ± s.d. (n = 3 technical replicates). Samples comparisons were performed using multiple t test corrected by the false discovery rate. * *P* < 0.05, ** *P* < 0.01, *** *P*< 0.001. **(E)** Secreted of IL-1β and IL-18 in cell supernatant were assayed with ELISA. Data were presented as mean ± s.d. (n = 3 technical replicates). Samples comparisons were performed using multiple t test corrected by the false discovery rate. * *P* < 0.05.


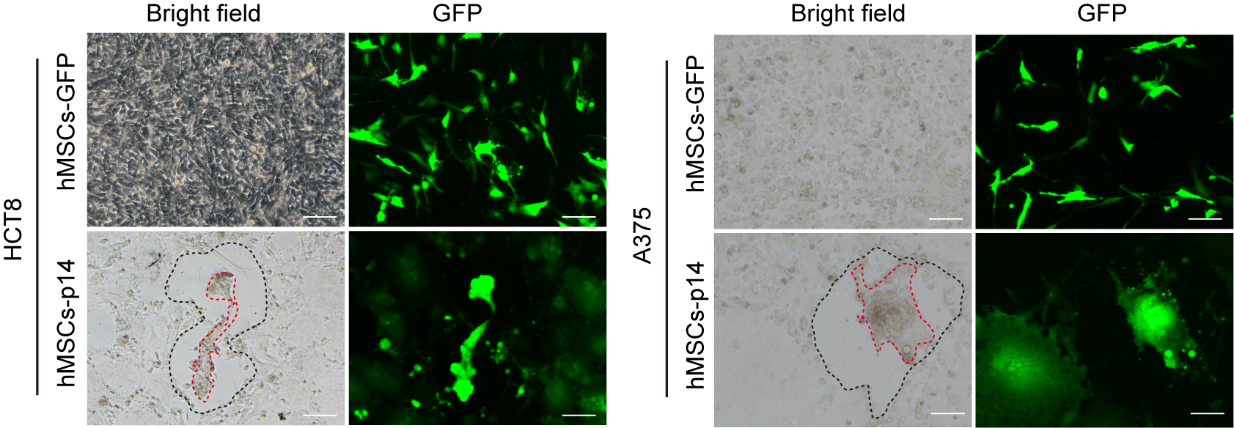


**Suppl. Figure 7 Syncytia were shrunk induced by MSCs-p14 at late stage of fusion.** Plasmids of PCDH-GFP or PCDH-p14-GFP were transfected into hMSCs and reacted for 8 hours. Subsequently, the engineered hMSCs were used as effector cells and cocultured with HCT8 and A375 cells. Syncytia continuously fused with peripheral and distant cells and shrank at about 20 hours after coculture. Spread syncytia were circled by black dotted lines, shrunken syncytia were circled by red dotted lines. Scale bar was 100 μm.


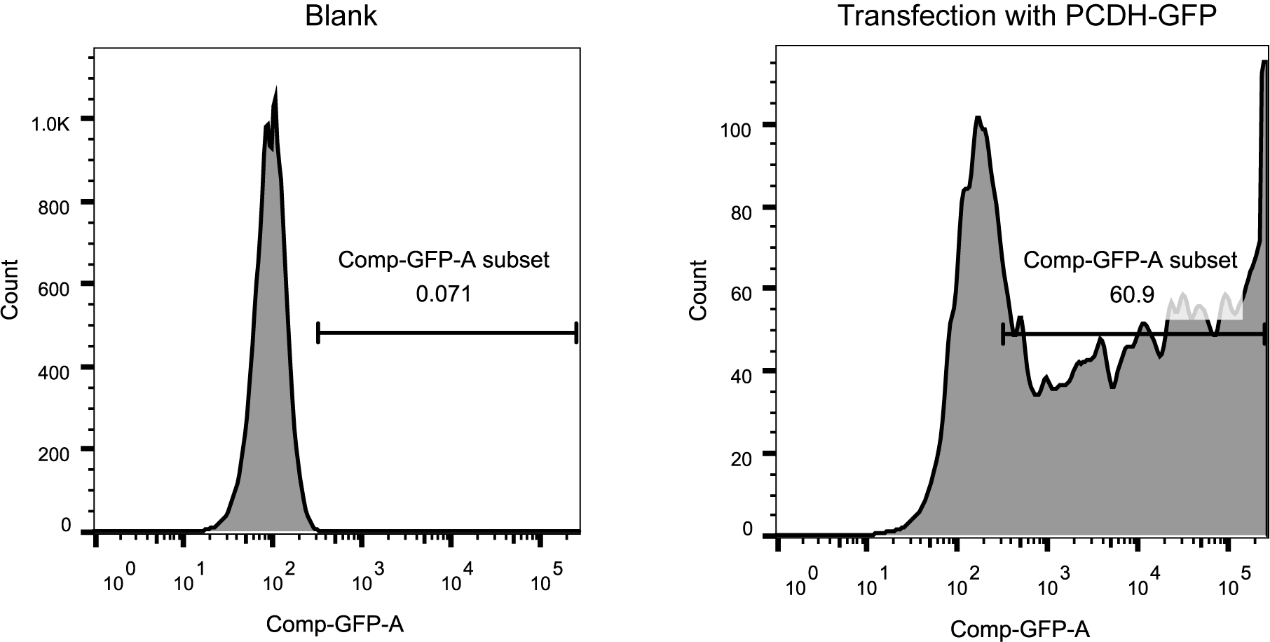


**Suppl. Figure 8. Transfection efficiency assay of PCDH-GFP-transfected MSCs.** Mouse MSCs were transfected with plasmid of PCDH-GFP by jetOPTIMUS. Twenty hours post-transfection, the GFP-positive cells were analyzed by flow cytometry.


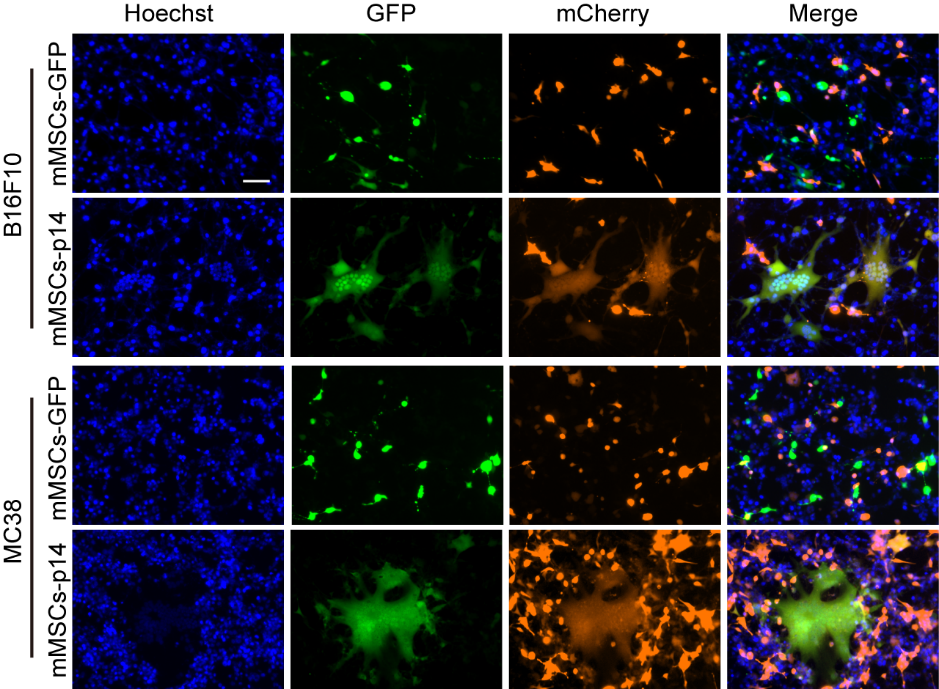


**Suppl. Figure 9. Mouse MSCs expressed p14 protein fused with mouse cancer cells of B16F10 and MC38.** Mouse MSCs as effector cells were transfected with plasmids of PCDH-GFP (mMSCs-GFP) or PCDH-GFP-p14 (mMSCs-p14) by jetOPTIMUS reagent. Eight hours post-transfection, the effector cells were mixed with MC38 and B16F10 cells expressing mCherry protein, and cocultured for another 24 hours. Nuclei was stained with Hoechst33342. Scale bar was 100 μm.


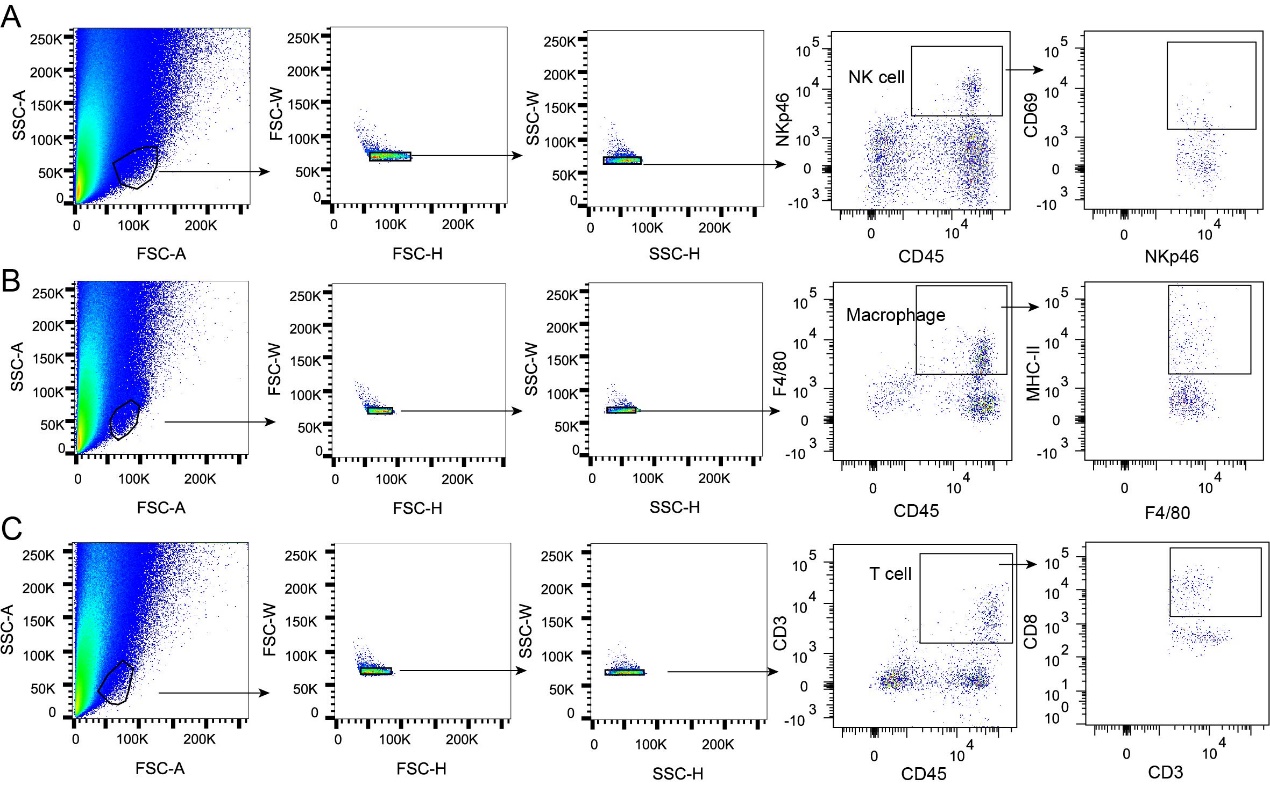


**Suppl. Figure 10 Flow cytometry gating strategy.** To determine whether immune cells exert anti-tumor effect in the late stage of cell fusion, we analyzed the intratumoral immune cells by flow cytometry. Live cells were gated on FSC-A/SSC-A, FSC-H/FSC-W, and then SSC-H/SSC-W. (A) NK cells were defined as CD45^+^NKp46^+^, CD69^+^ NK cells were defined as NKp46^+^CD69^+^ (A, for Figure 6A and B). (B) Macrophages were defined as CD45^+^F4/80^+^, MHC-II^+^ macrophage were defined as F4/80^+^ MHC-II^+^ (B, for Figure 6C and D). (C) T cells were defined as CD45^+^CD3^+^, CD8^+^ T cells were defined as CD3^+^CD8^+^ (C, Figure 6E and F).

**Suppl. Table 1 Primers of real-time PCR**

| **Name** | **Sequences (5’-3’)** |
| --- | --- |
| **hGAPDH-F** | CTCCTGCACCACCAACTGCT |
| **hGAPDH-R** | GGGCCATCCACAGTCTTCTG |
| **PMAIP-F** | GCTGGAAGTCGAGTGTGCTA |
| **PMAIP-R** | GAAACGTGCACCTCCTGAGA |
| **FOS-F** | TGCCTGCAAGATCCCTGATG |
| **FOS-R** | TGCTGCTGATGCTCTTGACA |
| **NFKBIA-F** | TGAAGTGTGGGGCTGATGTC |
| **NFKBIA-R** | GCTCGTCCTCTGTGAACTCC |
| **Gadd45B-F** | CCACTTCACGCTCATCCAGT |
| **Gadd45B-R** | TCGTGACCAGGAGACAATGC |
| **P14-F** | ATGGGCAGCGGACCTTCTAAC |
| **P14-R** | TTAGATGGCGGACACGTTGTC |
| **Human IFN-β F** | ACGCCGCATTGACCATCTAT |
| **Human IFN-β R** | GTCTCATTCCAGCCAGTGCT |
| **Human TNF-α F** | ACCTCCTCTCTGCCATCAAGA |
| **Human TNF-α R** | TCCCAAAGTAGACCTGCCCA |
| **Human IL-1β F** | GGCCCTAAACAGATGAAGTGCT |
| **Human IL-1β R** | TGAAGCCCTTGCTGTAGTGG |
| **human IL-12A F** | CTCCAGAAGGCCAGACAAAC |
| **Human IL-12A R** | CCAGGCAACTCCCATTAGTT |
| **Human IL-23A F** | CCAGCTTCATGCCTCCCTAC |
| **Human IL-23A R** | TTGAAGCGGAGAAGGAGACG |
